# Fine mapping of a quantitative trait locus for spikelet number per panicle in a new plant type rice and evaluation of a near-isogenic line for grain productivity

**DOI:** 10.1093/jxb/erx128

**Published:** 2017-06-03

**Authors:** Kazuhiro Sasaki, Daisuke Fujita, Yohei Koide, Patrick D Lumanglas, Ritchel B Gannaban, Analiza G Tagle, Mitsuhiro Obara, Yoshimichi Fukuta, Nobuya Kobayashi, Tsutomu Ishimaru

**Affiliations:** 1 Japan International Research Center for Agricultural Sciences (JIRCAS), Ohwashi, Tsukuba, Ibaraki, Japan; 2 International Rice Research Institute (IRRI), DA, Metro Manila, Philippines; 3 Graduate School of Agricultural and Life Sciences, Institute of Sustainable Agro-ecosystem Services (ISAS), The University of Tokyo, Midoricho, Nishitokyo, Tokyo, Japan; 4 Faculty of Agriculture, Saga University, Honjo-machi, Saga, Japan; 5 Research Faculty of Agriculture, Hokkaido University, Kita-9 Nishi-9, Kita-ku, Sapporo, Japan; 6 National Institute of Crop Science (NICS), NARO, Kannondai, Tsukuba, Ibaraki, Japan; 7 Central Region Agricultural Research Center (CARC), National Agriculture and Food Research Organization (NARO), Inada, Joetsu, Niigata, Japan

**Keywords:** Grain yield, Indica Group, near-isogenic line, *Oryza sativa* L, quantitative trait locus, total spikelet number per panicle, yield component

## Abstract

Total spikelet number per panicle (TSN) is one of the determinants of grain productivity in rice (*Oryza sativa* L.). In this study, we attempted to detect quantitative trait loci (QTLs) for TSN in the introgression lines with high TSN, derived from the cross of Indica Group variety IR 64 with new plant type lines. Two QTLs were detected on the long arm of chromosome 12: *qTSN12.1* in the BC_4_F_2_ population of YTH63/IR 64 and *qTSN12.2* in the BC_4_F_3_ population of YTH83/IR 64. TSN of the main tiller was significantly higher in near-isogenic lines (NILs) for *qTSN12.1* (IR 64-NIL1; 188.6) and for *qTSN12.2* (IR 64-NIL12; 199.4) than in IR 64 (141.2), owing to a significant increase in both primary and secondary branch numbers. These results suggest the critical function of these QTLs in the promotion of rachis branching at the panicle formation stage. Fine mapping of *qTSN12.2* revealed six candidate genes in a 92-kb region of the Nipponbare reference genome sequence between flanking markers RM28746 and RM28753. Detailed phenotyping of agronomic traits of IR 64-NIL12 carrying *qTSN12.2* showed drastic changes in plant architecture: this line had lower panicle number, longer culm, and longer and wider leaves compared with IR 64. Percentage of fertility and 1000-grain weight tended to be greater, and grain yield per square meter was also greater in IR 64-NIL12 than in IR 64. The newly identified QTLs will be useful for genetic improvement of the yield potential of Indica Group varieties. The markers tightly linked to *qTSN12.2* are available for marker-assisted breeding.

## Teaser

*qTSN12.1* and *qTSN12.2* genes for total spikelet number per panicle were detected, *qTSN12.2* on rice chromosome 12. Grain yield of a near-isogenic line carrying fine-mapped *qTSN12.2* was increased by 18–36%.

## Introduction

Rice (*Oryza sativa* L.) is an important crop that serves as the staple food of more than half the world’s population. To satisfy the increasing global demand of the growing population, a 50% increase in rice production will be required by the year 2050 (Alexandratos and Bruinsma, 2012), especially in developing countries of Asia and Africa, where populations have been increasing dramatically (Seck *et al.*, 2012). Of the two main subspecies of cultivated rice, the Indica Group varieties are grown predominantly in southern China, Southeast Asia, and South Asia, occupying about 70% of the world’s rice-producing area. The Japonica Group varieties, on the other hand, are grown mainly in East Asia (Zhao *et al.*, 2011; Huang *et al.*, 2012). Considering their vast area of production, the development of Indica Group varieties with high yield potential will contribute to global food security.

Rice yield is characterized by total spikelet number per panicle (TSN), panicle number, fertility, and 1000-grain weight. TSN is a key factor directly associated with rice productivity; hence, many studies have been conducted to identify genetic factors for TSN and elucidate its contribution to genetic improvement of yield potential (see review by Bai *et al.*, 2012). Quantitative trait loci (QTLs) for TSN in rice have been identified in various segregating populations (Bai *et al.*, 2012). In addition to QTLs for TSN, QTLs for controlling panicle architecture, such as the number of primary and secondary branches and spikelet number per primary and secondary branch, have also been mapped and studied (Yamagishi *et al.*, 2002; Ando *et al.*, 2008). Three of the QTLs associated with both TSN and panicle architecture, namely *qTSN4.4/SPIKE/LSCHL4* (Fujita *et al.*, 2012, 2013; Zhang *et al.*, 2014), *SCM2/APO1* (Ookawa *et al.*, 2010; Terao *et al.*, 2010), and *DEP1* (Huang *et al.*, 2009), were found to increase grain yield in near-isogenic lines (NILs) through drastic changes in plant architecture under field conditions.

In the late 1980s, a breeding program to develop new plant type (NPT) lines was launched at the International Rice Research Institute (IRRI) with the aim of increasing the yield potential of Indica Group inbred varieties in a tropical environment. The ideotypes of NPT would have low tiller number, few unproductive tillers, 200–250 grains per panicle, and increased harvest index (Khush, 1995). The IRRI breeding programs used Tropical Japonica Group varieties as donors; these varieties had low tillering, few unproductive tillers, large panicles, thick culms, lodging resistance, and large dark green ﬂag leaves (Khush, 1995). The developed NPT lines had higher TSN and 1000-grain weight, and a lower number of unproductive tillers; however, the NPT lines did not yield more grain because of poor fertility and fewer panicles (Peng *et al.*, 2008).

In the 1990s, a breeding objective was set under the IRRI-Japan Collaborative Research Project to improve the yield potential of the Indica Group variety IR 64 by using NPT lines and a Japanese high-yielding variety, Hoshiaoba, as donor parents. A total of 334 introgression lines (ILs, BC_3_-derived lines) with unique agronomic traits (high TSN, earlier or later heading date, and greater 1000-grain weight, leaf size, and culm length) were developed in the IR 64 genetic background by recurrent backcross breeding (Fujita *et al.*, 2009, 2010). Fujita *et al.* (2009) used these ILs to map chromosomal regions that were associated with favorable agronomic traits, including TSN. Using BC_4_F_2_ populations derived from the crosses between the ILs and IR 64, six QTLs for TSN were detected: five on chromosome 4, *qTSN4.1*–*qTSN4.5* (Fujita *et al.*, 2012), and one on chromosome 7, *qTSN7.1* (Koide *et al.*, 2013). The allelic effects of these QTLs were confirmed in NILs. Among the 334 ILs, a few lines had considerably high TSN but lacked segments containing *qTSN4*s and *qTSN7.1* (Fujita *et al.*, 2010). Therefore, we hypothesized that as yet unidentified QTLs for TSN are present on other chromosomes.

This study was conducted to explore new QTLs for TSN in two ILs with high TSN, YTH63 and YTH83, selected from among the 334 ILs. Two QTLs for TSN were identified and one of them was fine-mapped. NILs carrying QTLs for TSN were developed and used to investigate grain yield, yield components, and other agronomic traits under field conditions.

## Materials and methods

### Plant material

The ILs YTH63 and YTH83 were derived from the crosses of IR 64 with two NPT lines, IR 65598-112-2 and IR 65564-2-2-3, respectively (Table 1). YTH63 had neither *qTSN4* (Fujita *et al.*, 2012) nor *qTSN7* (Koide *et al.*, 2013) (see Supplementary Fig. S1A at *JXB* online), whereas YTH83 had *qTSN4.2* (Fujita *et al.*, 2012) (Supplementary Fig. S1B). Both ILs had introgressed segments on the short arm of chromosome 1 and long arm of chromosome 12 (Table 1 and Supplementary Fig. S1). YTH63 and YTH83 were crossed with IR 64 to generate BC_4_F_2_ populations for QTL analysis (Supplementary Fig. S2). In the BC_4_F_2_ population of YTH83 and IR 64, one QTL peak was found on chromosome 4 and another one on chromosome 12. To clarify the effect of the QTL on chromosome 12, we selected a BC_4_F_2_ plant that was heterozygous on chromosome 12 and IR 64-fixed homozygous on chromosome 4, and a total of 201 BC_4_F_3_ plants were generated by selfing from the BC_4_F_2_ plants and used for QTL analysis (see Supplementary Fig. S2).

**Table 1.** Plant materials in this study

Introgression line		Donor^*a*^		Parental varieties of donor^*b*^	Chromosomal location of introgressed segments^*c*^
Entry no.	IRRI accession no.	Entry no.	IRRI accession no.		
YTH63^*c*^	IR 84635-10-59-4-2-2-3-4-2-2-8-B	YP3	IR 65598-112-2	Shen Nung 89–366, **Genjah Wangkal**	1S, 12L
YTH83^*c*^	IR 84642-8-4-3-4-4-2-4-2-2-6-B	YP4	IR 65564-2-2-3	NO 11, **Bali Ontjer**	1S, 1L, 4L, 12L

^*a*^ Donors are NPT lines bred at IRRI.

^*b*^ Pedigrees obtained from the International Rice Information System (IRIS; http://www.iris.irri.org/). Bold indicates varieties from Indonesia (Tropical Japonica Group).

^*c*^ Information from Fujita *et al.* (2010). Refer to Supplementary Fig. S1 for graphical genotype.

### Plant growth conditions and panicle sampling

IR 64, YTH63, YTH83 and 185 BC_4_F_2_ plants of YTH63/IR 64 and 201 BC_4_F_3_ plants of YTH83/IR 64 were grown in an experimental paddy field in IRRI, Los Baños, the Philippines (14°11′N, 121°15′E). Soaked seeds were sown in trays containing sterilized fine soil and placed in a greenhouse. At 21 days after sowing, seedlings (one plant per hill) were transplanted at 20 cm between hills and 30 cm between rows. Basal fertilizer (30 kg ha^–1^ each of N, P_2_O_5_, and K_2_O) was applied as dressing before transplanting. At 2 and 4 weeks after transplanting, ammonium sulfate was applied at 30 kg N ha^–1^ as top dressing.

In the BC_4_F_2_ population (YTH63/IR 64), panicles of the three tallest culms of each individual plant were collected for counting TSN; the average TSN values were used for segregation analysis. In the BC_4_F_3_ population (YTH83/IR 64), the panicles of the tallest culm were harvested for counting TSN.

### QTL analysis

Genomic DNA from individual plants in each of the BC_4_F_2_ and BC_4_F_3_ populations was extracted from freeze-dried leaves using the cetyltrimethylammonium bromide (CTAB) method (Rogers and Bendich, 1985). The DNA samples were analyzed with simple sequence repeat (SSR) markers corresponding to known introgressed regions in each IL (Fujita *et al.*, 2010; McCouch *et al.*, 2002). PCR amplification and electrophoresis were conducted by the method described by Fujita *et al.* (2012). A linkage map with SSR markers located on all introgression segments was constructed based on genotypes of BC_4_F_2_ individuals in each population by using the Kosambi function (Kosambi, 1943). Composite interval mapping was performed using Windows QTL Cartographer V2.5 (Wang *et al.*, 2010). The proportion of observed phenotypic variations attributable to a particular chromosomal region was estimated by the coefficient of determination (*R*^*2*^). The critical threshold values of the LOD score for QTL identification were calculated by conducting 1000 permutation tests with significance at *P*<0.05.

### Substitution mapping

The development scheme for plant materials and populations used for QTL analysis and substituting mapping in this study are shown in Supplementary Fig. S2. Seven recombinants between RM28621 and RM1226 were selected from BC_4_F_3_ plants for the first substitution mapping. A total of 24 BC_4_F_4_ progeny from each selected BC_4_F_3_ plant (named HFO entry) were grown. At least three homozygous recombinant plants in each BC_4_F_4_ progeny were selected for TSN evaluation and estimation of the genotype for the QTL mapping. Primers designed using the genome sequence of Nipponbare (Rice Genome Research Program; http://rapdb.dna.affrc.go.jp/) are listed in Supplementary Table S1. Using these primers, additional recombinant BC_4_F_3_ plants from a large population (5841 plants) were selected. For the second substitution mapping, a total of 47 recombinants between KM12016 and RM17 (named MBV entry) were selected to narrow down the candidate QTL region. Selfing of these selected plants produced homozygous recombinant BC_4_F_4_ plants. To determine the genotype for the QTL in the BC_4_F_4_ recombinants, 24 BC_4_F_5_ progeny and IR 64 were grown in the paddy field. Plant growth conditions both for the first and second substitution mapping were the same as described in ‘Plant growth conditions and panicle sampling’. One panicle was harvested per plant from the tallest culm for TSN counting.

### NIL development

One plant containing the detected QTL for TSN and the smallest number of introgressed segments was selected from the BC_4_F_2_ (YTH63/IR 64) population, and was used to generate BC_4_F_3_ lines by self-pollination. A NIL selected from the BC_4_F_3_ lines was designated IR 64-NIL1. One plant was selected from the BC_4_F_3_ (YTH83/IR 64) population with the same method and designated IR 64-NIL12. (IR 64-NILs were numbered in chronological order as they were developed under the IRRI-Japan Collaborative Research Project.) The NILs were grown during the dry season (DS) of 2009 in IRRI. One panicle in the tallest culm of each of at least nine plants was collected for counting the numbers of primary branches, secondary branches, tertiary branches, number of spikelets on each branch, and TSN. The numbers of primary, secondary, and tertiary branches were counted according to the rice panicle structure described by Ikeda *et al.* (2010).

### Gene expression analysis and sequencing of a candidate gene

Quantitative RT-PCR (qRT-PCR) for annotated genes located in the region of *qTSN12.2* was performed. IR 64 and IR 64-NIL12 were grown in an experimental paddy field of NICS, Ibaraki, Japan (36°00′N, 140°01′E) in 2016. Note that increased TSN in IR 64-NIL12 was stably observed also in this experimental paddy field (see Supplementary Table S2). Three developing panicles (2–5 mm) were collected as one biological replicate from a plant at panicle initiation stage, and four biological replicates (four plants) were prepared for this expression analyses in both genotypes. Total RNA was extracted by using an RNeasy Plant Mini Kit (Qiagen, Hilden, Germany). One microgram of total RNA was used for the reverse transcriptase reaction (Prime Script RTase, Takara Bio Inc., Otsu, Japan). qRT-PCR reactions were carried out with 1.5 μl cDNA mixtures under a Thermal Cycler Dice Real Time System III (Takara Bio). The expression level of annotated genes was normalized to the expression of a household gene, ubiquitin (Os01g22490), in the developing panicles (Fujita *et al.*, 2013). The expression of each gene was compared between IR 64 and IR 64-NIL12 using the Δ Δ *C*_t_ method (Livak and Schmittgen, 2001). All statistics were performed at the Δ *C*_t_ stage with the *t*-test.

### Grain yield, shoot biomass, and harvest index

IR 64 and IR 64-NIL12 were cultivated for yield measurements in the wet season (WS) of 2011 and 2012, and in the 2012 DS and 2013 DS in IRRI. Three germinated seeds were sown into each cell of a cell tray and grown in the greenhouse under natural light conditions for 3 weeks. Seedlings (three per hill) were transplanted at 20 cm between hills and 25 cm between rows. The area of each plot was 4.8 m^2^, with three or four replications. The experimental plots were arranged in a complete randomized block design. Basal and top dressings were applied as described above. At maturity, 20 plants corresponding to 1 m^2^ were harvested at the soil surface in each plot. Grain and straw were dried in a greenhouse. Grain yield was calculated on a 14% moisture content basis.

### Yield components and agronomic traits

Yield components and agronomic traits in IR 64 and IR 64-NIL12 were measured in 2011 WS and 2012 DS in IRRI. Days to heading was determined as the number of days from seeding to 50% ﬂowering. Panicle number, TSN, percentage fertility, and 1000-grain weight were determined at maturity from five plants selected from each plot of the yield measurements. After being removed from panicles, approximately 30 g of grains was randomly selected as subsamples to determine total spikelet number per square meter, fertility, and 1000-grain weight. Sterile and fertile grains were manually selected and 1000-grain weight of fertile grain was calculated on a 14% moisture content basis. Culm length, panicle length, leaf length, and leaf width were measured on the tallest tiller of each plant, with culm length measured from the soil surface to the panicle neck, panicle length measured from the panicle neck to the panicle tip, and leaf width and leaf length measured on the second leaf (i.e. the one below the ﬂag leaf).

## Results

### QTL analysis for TSN

**Fig. 1.**
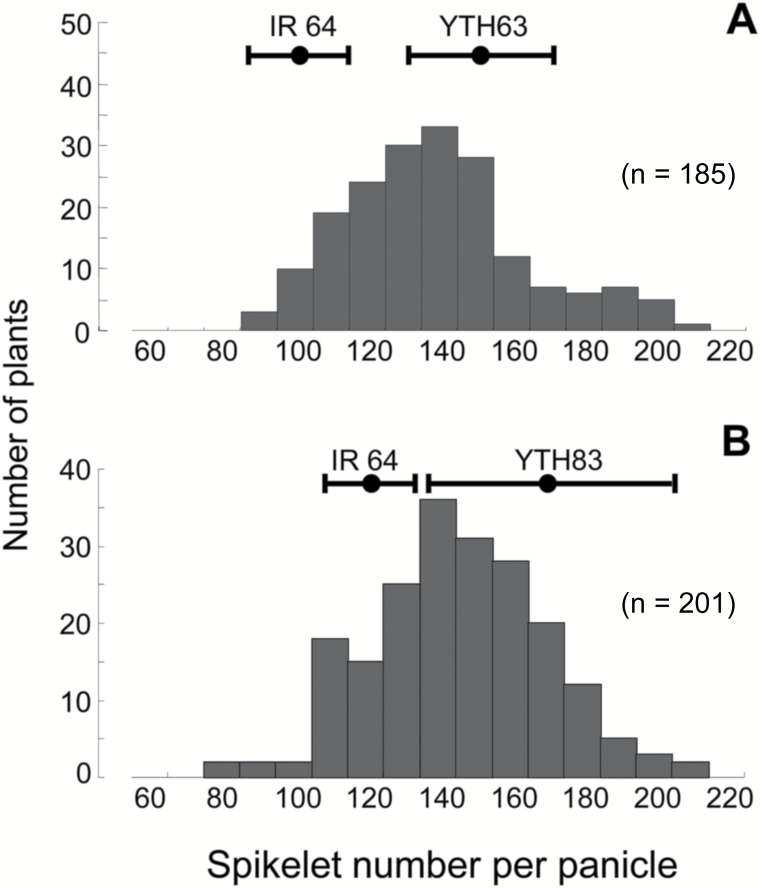
Frequency distribution of total spikelet number per panicle in BC_4_F_2_ and BC_4_F_3_ populations derived from crosses of IR 64 with (A) YTH63 or (B) YTH83.

**Fig. 2.**
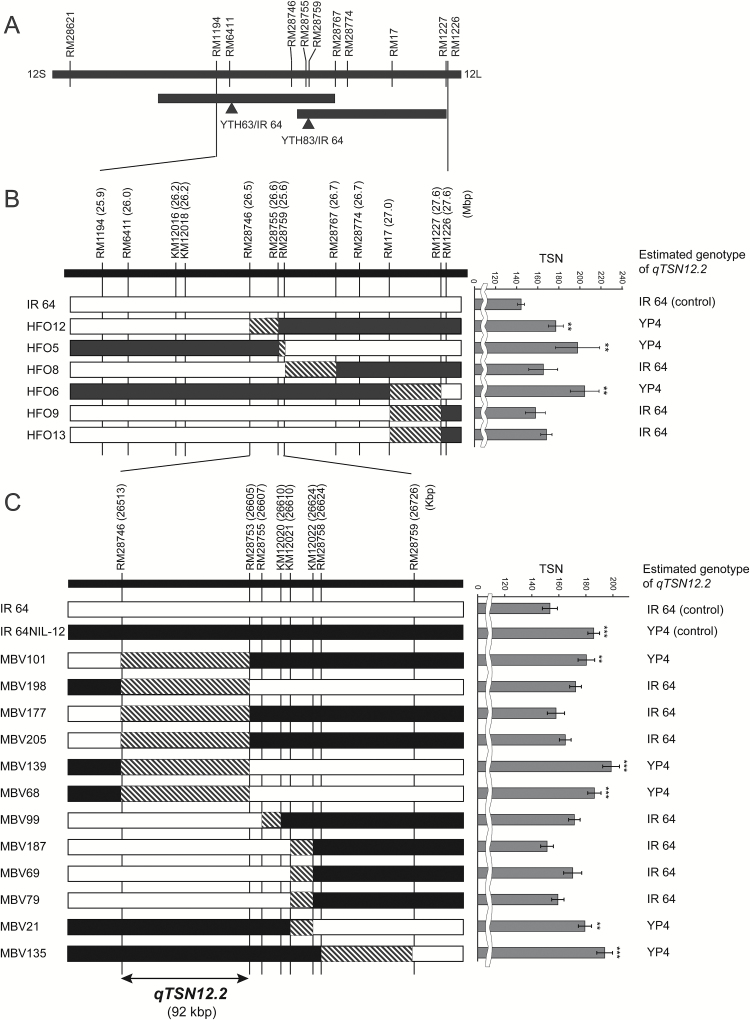
Mapping of QTL for total spikelet number per panicle (TSN) detected on chromosome 12 in the BC_4_F_2_ population of YTH63/IR 64 (*qTSN12.1*) and the BC_4_F_3_ population of YTH83/IR 64 (*qTSN12.2*) and the location of *qTSN12.2*. (A) QTL analysis. Black bars indicate QTL regions. Arrowheads indicate the peak position of each QTL. (B, C) The first (B) and the second (C) substitution mapping. Graphical genotypes and total spikelet number per panicle (TSN) of the BC_4_F_3_ plants (YTH83/IR 64). Black, homozygous for YTH83; white, homozygous for IR 64; hatched, recombination regions. Positions of DNA markers are indicated by vertical lines. The TSN data are mean values with error bars indicating standard errors. Double-headed arrow indicates the *qTSN12.2* candidate region.

TSN of YTH63 was 148.3 ± 19.3 (mean±standard deviation), which was considerably higher than that of IR 64 (98.2 ± 14.4) (Fig. 1A). TSN in the BC_4_F_2_ (YTH63/IR 64) population showed a continuous frequency distribution and ranged from 87.7 to 202.3 (Fig. 1A). Composite interval mapping identified a QTL in YTH63 between RM6411 and RM28746 on the long arm of chromosome 12 (Fig. 2A); this QTL, designated as *qTSN12.1*, explained 10.0% of phenotypic variation (Table 2). The donor allele at *qTSN12.1* increased TSN (Table 2). TSN of YTH83 was 177.2 ± 41.6, which was considerably higher than that of IR 64 (121.1 ± 17.2) (Fig. 1B). TSN in the BC_4_F_3_ (YTH83/IR 64) population showed a continuous frequency distribution and ranged from 76 to 209 (Fig. 1B). The QTL in YTH83 was between RM28759 and RM28767 on the long arm of chromosome 12 (Fig. 2A) and was designated as *qTSN12.2*; it explained 7.0% of phenotypic variation (Table 2). The donor allele at *qTSN12.2* increased TSN (Table 2).

**Table 2.** Quantitative trait loci (QTL) for total spikelet number in two populations

Cross combination	Donor	QTL	Marker interval^*a*^	Chr.	LOD score^*b*^	*R* ^2*c*^	Additive effect^*d*^	Dominant effect
YTH63/IR 64	YP3	*qTSN12.1*	**RM6411**–RM28746	12	4.2	0.10	–9.1	8.5
YTH83/IR 64	YP4	*qTSN12.2*	**RM28759**–RM28767	12	3.1	0.07	–10.1	6.2

^*a*^ Bold indicates nearest marker to QTLs.

^*b*^ Critical threshold value of LOD score was equivalent to LOD at an experiment-wise significant level of 0.05.

^*c*^ Percentage of explained phenotypic variation.

^*d*^ Positive value indicates the direction of the effect of IR 64 allele.

### Delimitation of a candidate genomic region for qTSN12.2

Among 201 BC_4_F_3_ plants (YTH83/IR 64), six that had recombination events between RM1194 and RM1227 were selected (Fig. 2B). The TSN values of HFO5, 6 and 12 were significantly higher than that of IR 64, whereas the TSN values of HFO8, 9 and 13 were slightly but not significantly higher than that of IR 64 (Fig. 2B). *qTSN12.2* was mapped within the interval between RM28746 and RM28759 as a single Mendelian factor (Fig. 2B). For more precise determination of the position of *qTSN12.2*, we selected additional recombinant BC_4_F_3_ plants from a large population. The TSN values of MBV198, 177, 205, 99, 187, 69, and 79 were similar to that of IR 64, whereas the TSN values of MBV101, 139, 68, 21, and 135 were significantly higher than that of IR 64. These results strongly indicate that *qTSN12.2* is located in the interval between RM28746 and RM28753. According to the Nipponbare genome sequence in the Rice Annotation Project Database (2015), the physical distance of this interval is approximately 92 kb (Fig. 2C). Furthermore, quantitative gene expression analysis was performed for open reading frames (ORFs) located in this candidate region using primer pairs described in Table 3. Of nine putative ORFs, primer pairs for Os12g0619100 and Os12g0620100 could not be developed possibly due to the negligible expression level in the various rice organs, according to the RNA-seq analysis in the Rice Annotation Project Data Base (RAP-DB, 2015). Expression of Os12g0619700 was not detectable in RT-PCR analysis. Among the remaining six putative ORFs, no gene was significantly higher in IR 64-NIL12 than IR 64 (Table 3), although expression of Os12g0619000 was up-regulated by 27% in IR 64-NIL12 relative to IR 64 (Table 3).

**Table 3.** Relative expression and primer information on annotated genes in candidate region of qTSN12.2

Locus^*a*^	Annotation	Forward primer sequence (5′–3′)	Reverse primer sequence (5′–3′)	Relative expression (IR 64-NIL12/IR 64)^*b*^
Os12g0618800	Protein of unknown function DUF266, plant family protein	GCGAGCAGTTTGTTCACTCA	GGGATCGGATCTTGCTTACA	1.10 ns
Os12g0619000	IQ calmodulin-binding region domain containing protein	ATCAGTGGAGCCAGAATTGG	GGCCTCATTTTCATCAGCAT	1.27 ns
Os12g0619700	Conserved hypothetical protein	AACCTGTTTACTGCGGTTCG	AATGTCCCTGCAGAACCTTG	nd
Os12g0620000	Similar to leucine rich repeat family protein	AGAAGCATCCGCAGCTATGT	CGCCACTCTGAGAACTGACA	1.15 ns
Os12g0620400	Methyl-CpG DNA binding domain containing protein	GTCTCGTCTCTTCTCTCTCCATTTT	CATGGTTTCGAGTTTTCGCTTC	0.91 ns
Os12g0620600	Conserved hypothetical protein	TTGATTCTGGGTACCGCTTC	CCTAATTCCACCAGGCTCAA	0.82 ns
Os12g0621000	Similar to ubiquitin carboxyl-terminal hydrolase	CATTTGGGGATTTGTTGAGG	AGCTCCTGGGAATCATGTTG	0.90 ns

^*a*^ Os12g0619100 (heat shock protein DnaJ, cysteine-rich domain containing protein) and Os12g0620100 (zinc finger, RING-type domain containing protein) are also annotated in this region.

^*b*^ ns, the differences were not significant; nd, gene expression was not detected.

### Development of NILs and characterization of panicle architecture

**Fig. 3.**
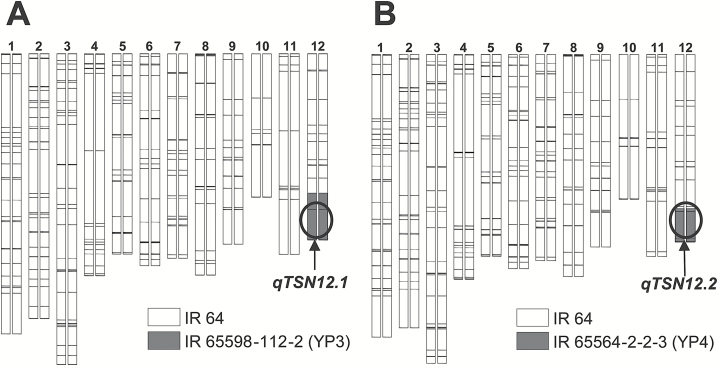
Graphical genotypes of near-isogenic lines. (A) IR 64-NIL1 for *qTSN12.1*; (B) IR 64-NIL12 for *qTSN12.2*. White and gray boxes indicate the segments derived from IR 64 and donor parents, respectively. Horizontal lines across the chromosomes indicate the positions of DNA markers. Circles indicate the approximate positions of *qTSN12.1* and *qTSN12.2*.

A BC_4_F_3_ line generated by selfing of the selected BC_4_F_2_ plants (YTH63/IR 64) only contained the region of *qTSN12.1*. This line was selected as a NIL for *qTSN12.1* and designated as IR 64-NIL1 (Fig. 3A). From the BC_4_F_3_ population of YTH83/IR 64, one BC_4_F_3_ plant containing the region of *qTSN12.2* was selected through the same method used for the development of IR 64-NIL1. A BC_4_F_4_ line generated by selfing of the selected BC_4_F_3_ plant was designated as IR 64-NIL12 (Fig. 3B). A chromosome segment of 3.96–6.28 Mbp from YP3 was carried on chromosome 12 of IR 64-NIL1; 3.96–4.05 Mbp of chromosome segment from YP4 was carried on chromosome 12 of IR 64-NIL12. In both cases, the interval of chromosome segment from donor parent is between RM1102 and RM1227.

To confirm the effects of *qTSN12.1* and *qTSN12.2* on TSN, spikelets were counted separately on the primary and secondary branches of the NILs. The TSN was 188.6 ± 6.8 in IR 64-NIL1 and 199.4 ± 9.1 in IR 64-NIL12, which were significantly higher than that of IR 64 (141.2 ± 5.6) (Table 4). The spikelet numbers on the primary rachis were 53.7 ± 1.2 in IR 64-NIL1 and 50.7 ± 1.6 in IR 64-NIL12, which were significantly higher than that of IR 64 (45.6 ± 1.0). The spikelet numbers on the secondary rachis were 134.9 ± 6.3 in IR 64-NIL1 and 148.7 ± 9.1 in IR 64-NIL12, which were also significantly higher than that of IR 64 (95.6 ± 5.3) (Table 4). The numbers of primary and secondary branches in the two NILs were also significantly higher than that of IR 64 (Table 4). The average spikelet number per branch located on the secondary rachis of the NILs was significantly higher than that of IR 64, but those on primary branches of the NILs and IR 64 were statistically similar (see Supplementary Table S3).

**Table 4.** *Panicle architecture of near-isogenic lines for* qTSN12.1, qTSN12.2 *and IR 64 in the dry season of 2009*

Line	QTL	Spikelet no. per panicle	No. of spikelet		No. of branch	
			Primary branch	Secondary branch	Primary	Secondary
IR 64	—	141.2 ± 5.6	45.6 ± 1.0	95.6 ± 5.3	9.2 ± 0.1	28.6 ± 1.3
IR 64-NIL1	*qTSN12.1*	188.6 ± 6.8***	53.7 ± 1.2***	134.9 ± 6.3**	10.8 ± 0.3***	38.4 ± 1.5***
IR 64-NIL12	*qTSN12.2*	199.4 ± 9.1***	50.7 ± 1.6*	148.7 ± 9.1***	10.1 ± 0.2*	40.4 ± 2.0***

At least nine plants in each line were investigated; the mean and standard deviation are shown. All means for the NILs differed significantly from those for IR 64 at the 5% (*), 1% (**) or 0.1% (***) levels (Dunnett’s test).

### Grain yield, shoot biomass, and harvest index

**Fig. 4.**
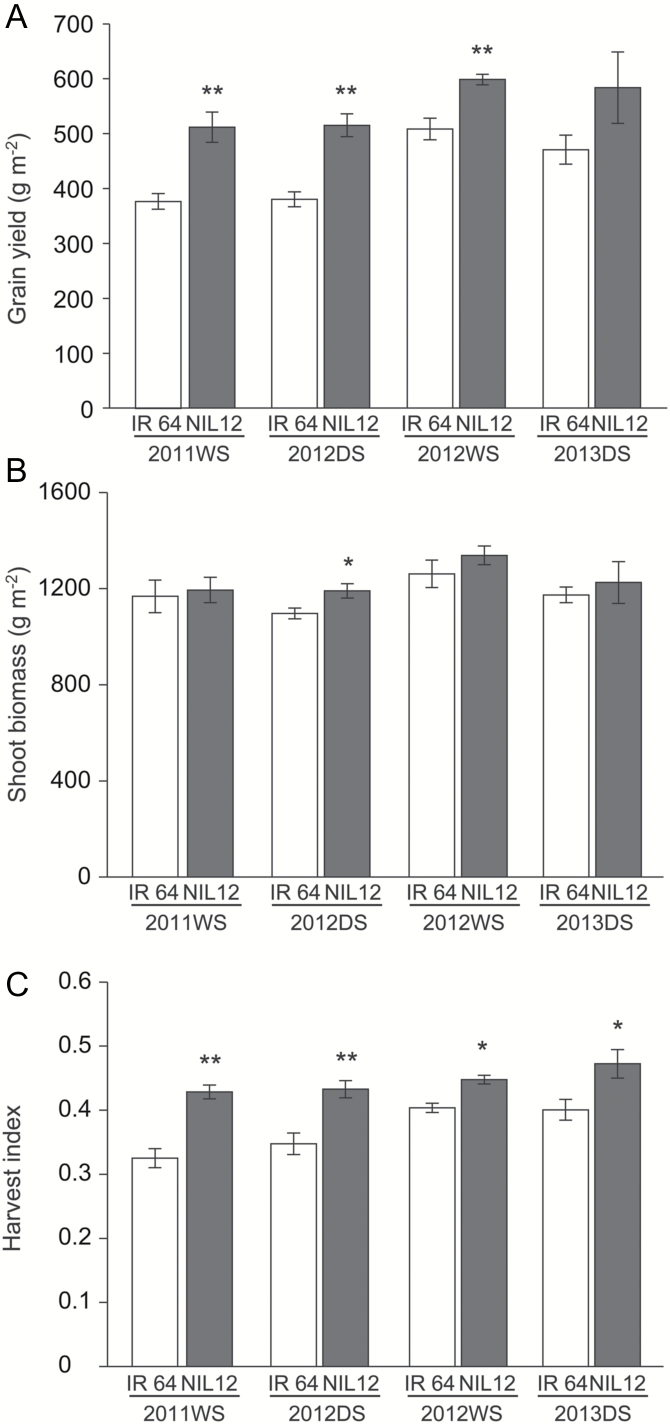
Characteristics of near-isogenic line IR 64-NIL12. (A) Grain yield, (B) shoot biomass and (C) harvest index of IR 64 and IR 64-NIL12 in the wet seasons of 2011 and 2012, and the dry season of 2012 and 2013. * and ** indicate significant differences between lines at the 5% or 1% level according to the *t*-test. Error bars show standard error (*n* = 3 or 4).

IR 64-NIL12 was selected for yield measurements because it had a smaller segment introgressed from the donor parent on the long arm of chromosome 12 than in IR 64-NIL1. Grain yield per square meter ranged from 380 to 508 g m^–2^ in IR 64 and from 512 to 598 g m^–2^ in IR 64-NIL12 (Fig. 4A). Grain yield per square meter was higher in IR 64-NIL12 by 18–36% than in IR 64, and this difference was significant for three of the four seasons (Fig. 4A). Shoot biomass was similar between IR 64 and IR 64-NIL12 in three of the four seasons (Fig. 4B). Harvest index of IR 64-NIL12 was significantly higher (by 11–32%) than that of IR 64 in all four seasons (Fig. 4C).

### Yield components and agronomic traits

Agronomic traits (days to heading, culm length, panicle length, leaf length, and leaf width) measured in 2011 WS and 2012 DS are shown in Table 5. IR 64-NIL12 had longer culms and longer and wider leaves than IR 64 in both seasons. There were no significant differences in days to heading. These results indicate that the region of *qTSN12.2* increases plant height and leaf size without any changes in days to heading. Panicle length was similar between genotypes.

**Table 5.** Agronomic traits for IR 64 and near-isogenic line IR 64-NIL12 in the wet season of 2011 and dry season of 2012

Season		Line	Days to heading	Culm length (cm)	Panicle length (cm)	Leaf length (cm)	Leaf width (cm)
2011	Wet	IR 64	84.8 ± 0.5	77.9 ± 1.7	25.1 ± 0.2	38.7 ± 0.9	1.36 ± 0.05
		IR 64-NIL12	85.3 ± 0.5 ns	81.3 ± 0.8 ns	26.1 ± 0.5 ns	45.2 ± 1.0**	1.53 ± 0.02*
2012	Dry	IR 64	82.8 ± 0.3	65.4 ± 0.4	23.2 ± 0.5	34.8 ± 0.9	1.20 ± 0.02
		IR 64-NIL12	82.5 ± 0.3 ns	72.3 ± 1.0**	23.9 ± 0.3 ns	39.3 ± 0.5**	1.38 ± 0.03**

Values are means±standard error. * or ** indicate that IR 64-NIL12 differed significantly from IR 64 at the 5% or 1% level, respectively (*t*-test). ns, difference not significant.

Yield components of IR 64 and IR 64-NIL12 were evaluated in the 2011 WS and 2012 DS (Table 6). TSN values in IR 64-NIL12 were significantly higher by 20% (2011 WS) and 75% (2011 WS) than that of IR 64. Panicle numbers in IR 64-NIL12 were significantly lower by 16% (2011 WS) and 26% (2012 DS) than those of IR 64. Tiller numbers of IR 64-NIL12 were also significantly lower than those of IR 64 in the period of most growth in both DS and WS (Supplementary Fig. S3). Fertility of IR 64-NIL12 was 9% (2011 WS) and 6% higher (2012 DS) than that of IR 64 and 1000-grain weights were 3% and 5% greater (Table 6); however, these differences were significant only in the DS.

## Discussion

### Detection of QTLs for TSN, qTSN12.1 and qTSN12.2, on chromosome 12

We previously detected QTLs for TSN on chromosome 4 (*qTSN4.1–qTSN4.5*, Fujita *et al.*, 2012) and chromosome 7 (*qTSN7.1*, Koide *et al.*, 2013). In this study, we hypothesized that undetected QTLs for TSN derived from NPT lines are also present on other chromosomes. Using populations derived from crosses between IR 64 and YTH63 and between IR 64 and YTH83, two such QTLs, *qTSN12.1* and *qTSN12.2*, were detected on the long arm of chromosome 12 (Fig. 2A and Table 2). In close proximity, *qGPP12*, a QTL associated with spikelet number, was detected in a population derived from a cross between Zhenshan 97 (Indica Group) and Teqing (Indica Group), in which the Teqing allele increased TSN (Liu *et al.*, 2011). Subsequently, we succeeded in developing two NILs, IR 64-NIL1 carrying *qTSN12.1* and IR 64-NIL12 carrying *qTSN12.2*, with IR 64 genetic backgrounds in this study (Fig. 3). In both NILs, TSNs of the main tiller were significantly higher than that of IR 64 (Table 4). The numbers of spikelets on both primary and secondary rachis branches were significantly higher in IR 64-NIL1 and IR 64-NIL12 than in IR 64 (Table 4), whereas the average spikelet number per branch in the NILs was similar to that of IR 64 (see Supplementary Table S3). Therefore, the greater TSN in the NILs was attributed to greater branching. It is concluded that the donor alleles of *qTSN12.1* and *qTSN12.2* promote branching in the primary and secondary rachis in the genetic background of IR 64.

We further fine-mapped *qTSN12.2* in the interval between RM28746 and RM28753 (Fig. 2B, C). Physical distance of the candidate region for *qTSN12.2* is 92-kb according to the Nipponbare reference genome sequence (Fig. 2C). This region contains nine putative ORFs. Among predicted genes in the candidate region of *qTSN12.2*, no gene showed significant difference in expression level between IR 64 and IR 64-NIL12, but our survey and analysis on gene expression narrowed down the candidate genes to six (Table 3). There is no report so far demonstrating a homologue of any of the listed six genes was involved in increase in TSN. The causal gene for *qTSN12.2* is possibly the novel gene controlling the TSN. Further genetic studies sequencing the six candidate genes to find polymorphisms and subsequent validation with transgenic plants are required to determine the causal gene for *qTSN12.2*.

**Table 6.** Yield components for IR 64 and near-isogenic line (IR 64-NIL12) in the wet season of 2011 and the dry season of 2012

Season		Line	Panicle no. (plant^–1^)	Panicle no. (m^–2^)	Total spikelet no. (panicle^–1^)	Total spikelet no. (m^–2^)	Fertility (%)	1000-grain weight (g)
2011	Wet	IR 64	16.7 ± 0.7	333.5 ± 14.7	68.4 ± 4.5	22721 ± 1390	74.8 ± 2.7	26.5 ± 0.2
		IR 64-NIL12	14.0 ± 0.6 (0.84)*	280.0 ± 11.2 (0.84)*	82.3 ± 2.9 (1.20)*	23055 ± 1337 (1.01) ns	81.6 ± 1.5 (1.09) ns	27.2 ± 0.3 (1.03) ns
2012	Dry	IR 64	21.1 ± 0.8	422.0 ± 16.2	46.9 ± 2.4	19860 ± 1519	84.1 ± 1.0	26.6 ± 0.3
		IR 64-NIL12	15.7 ± 0.7 (0.74)*	314.0 ± 14.1 (0.74)*	82.0 ± 2.5 (1.75)**	25765 ± 1425 (1.30)*	89.4 ± 0.9 (1.06)**	27.8 ± 0.3 (1.05)*

The values are mean ± standard error (proportion of the value for IR 64). * or ** indicate significant difference with IR 64 at the 5% or 1% level according to the *t*-test. ns, difference not significant.

### Drastic changes in plant architecture in IR 64-NIL12 carrying qTSN12.2

Under field conditions, IR 64-NIL12 showed not only higher TSN, but also significantly lower tiller numbers, longer culms and leaves, and wider leaves compared with IR 64 (Table 5 and Supplementary Fig. S3). Reduced panicle number at maturity (Table 6) was in agreement with a lower tiller number (Supplementary Fig. S3). These results imply that the introgression of the *qTSN12.2* region drastically changes agronomic traits in the above-ground organs of IR 64. *qGPP12* increases TSN through prolonged growth under long-day conditions (Liu *et al.*, 2011). Although the effects of *qTSN12.2* and *qGPP12* on TSN are similar (as mentioned in the previous subsection), the effects of these QTLs on growth duration seem to be different because number of days to heading was not affected by *qTSN12.2* (Table 5). QTLs for tiller (panicle) number were detected close to the *qTSN12.2* locus (Yan *et al.*, 1999; Liao *et al.*, 2001; Hittalmani *et al.*, 2003; Yang *et al.*, 2006; Liu *et al.*, 2012). A QTL for leaf width was also identified near this region (Yan *et al.*, 1999). There is a possibility that *qTSN12.2* could play a crucial role in modifying plant architecture both in sink organs (TSN and tiller (panicle) number) and source organs (leaf size). Further genetic study is needed to clarify whether agronomic traits pleiotropically changed in IR 64-NIL12 are under the control of a single causal gene or multiple genes independently in the region of *qTNS12.2*.

### Genetic improvement of yield potential in Indica Group varieties with the introgression of qTSN12.2 region from a New Plant Type variety

Grain yield per square meter was significantly higher in IR 64-NIL12 than in IR 64 in three of the four seasons (Fig. 4A). The TSN was also higher in IR 64-NIL12 than in IR 64 both in the 2011 WS and 2012 DS (Table 6), consistent with the results for TSN of the main stem (Table 4). These results indicate that the enhanced TSN by the donor allele of *qTSN12.2* is one of the critical factors for increasing grain yield in the IR 64 genetic background. Fertility and 1000-grain weight in the IR 64-NIL12 were higher in both 2011 WS and 2012 DS, and significantly so in 2012 DS (Table 6). Our results also indicate that the introgression of the *qTSN12.2* region positively changed fertility and 1000-grain weight. Greater TSN is often linked with lower fertility, lower 1000-grain weight, or both, which may be caused by severe competition for carbohydrate allocation among grains within a rachis, consequently causing poor grain filling (Nagata *et al.*, 2002). We previously reported that *SPIKE* increased the grain yield of IR 64 and drastically changed plant architecture (Fujita *et al.*, 2013). Although fertility was greater, 1000-grain weight was significantly lower in NIL-*SPIKE* than in IR 64 (Fujita *et al.*, 2013). Both *qTSN12.2* and *SPIKE* enhance TSN; the effect on 1000-grain weight is different. Larger leaf size in IR 64-NIL12 (Table 5) is likely advantageous in producing more photosynthate during grain filling (Zhang *et al.*, 2014). The greater harvest index (Fig. 4C) observed in IR 64-NIL12 was not due to a consistent increase in shoot biomass (Fig. 4B), meaning that a larger amount of dry matter is allocated to the panicles in IR 64-NIL12 than in IR 64 during grain filling. Further studies are needed to elucidate the effect of the *qTSN12.2* region on the physiological changes in the production and allocation of assimilates to each organ in relation to changes in plant architecture. Under the given growth conditions, IR 64-NIL12 carrying *qTSN12.2* achieved higher grain yield than IR 64 (Fig. 4A). The developed markers linked with *qTSN12.2* could be useful for the genetic improvement of yield potential of Indica Group varieties. Note, however, that panicle number was reduced due to the introgression of the *qTSN12.2* region (Table 6). Khush *et al*. (1995) described that high TSN is often associated with low panicle number. IR 64-NIL12 is not an exception to this trade-off relationship. To further support the genetic improvement in the yield potential of Indica Group varieties and to recover the reduction in panicle number observed in IR 64-NIL12 carrying *qTSN12.2*, introgression of positive QTLs for panicle number, located at a different locus from *qTSN12.2*, into IR 64-NIL12 is required as a genetic approach. The establishment of better fertilizer management to promote tillering in IR 64-NIL12 could be an effective agronomic approach.

## Supplementary data

Supplementary data are available at *JXB* online.

Fig. S1. Graphical genotypes of the introgression lines YTH63 and YTH83.

Fig. S2. Scheme of development for mapping populations and near-isogenic lines.

Fig. S3. Tiller numbers for IR 64 and IR 64-NIL12 grown in a paddy field in the wet season of 2011 and dry season of 2012.

Table S1. Molecular markers developed for substitution mapping.

Table S2. Total spikelet number per panicle (TSN) for IR 64 and IR 64-NIL12 in the experimental field of NICS, Japan, in 2011 and 2012.

Table S3. Number of spikelets on primary and secondary branches in IR 64 and near-isogenic lines carrying *qTSN12* in dry season of 2009.

## Funding

This study was financially supported by the Japanese government (IRRI-Japan Collaborative Research Project).

## Supplementary Material

Supplementary_Figures_S1_S3

Supplementary_Tables_S1_S3
