# Supplementary material for: Fine mapping of a quantitative trait locus for spikelet number per panicle in a new plant type rice and evaluation of a near-isogenic line for grain productivity
Source: J Exp Bot. 2017 Jun 3;68(11):2693–702. doi: 10.1093/jxb/erx128 (PMC5853308; doi:10.1093/jxb/erx128)
Supplement: Supplementary_Figures_S1_S3 [file erx128_suppl_supplementary_figures_s1_s3.pdf]

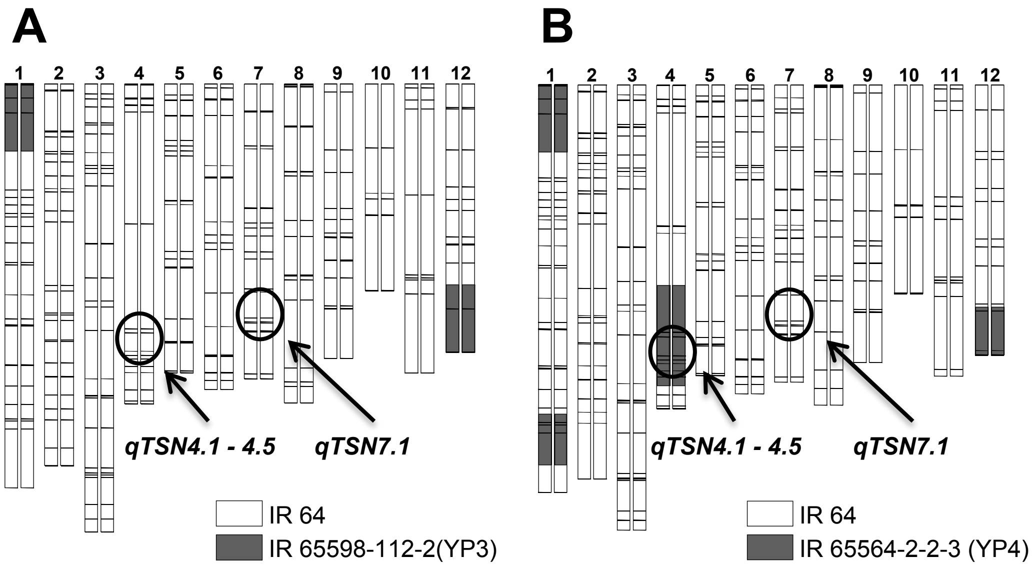

Supplementary Figure S1. Graphical genotypes of the introgression lines YTH63 (A) and YTH83 (B).

White and gray boxes indicate segments derived from IR 64 and donor parents, respectively. Horizontal lines across the chromosomes indicate the positions of DNA markers. Circles indicate the approximate positions of *qTSN4s* and *qTSN7.1* on chromosomes.

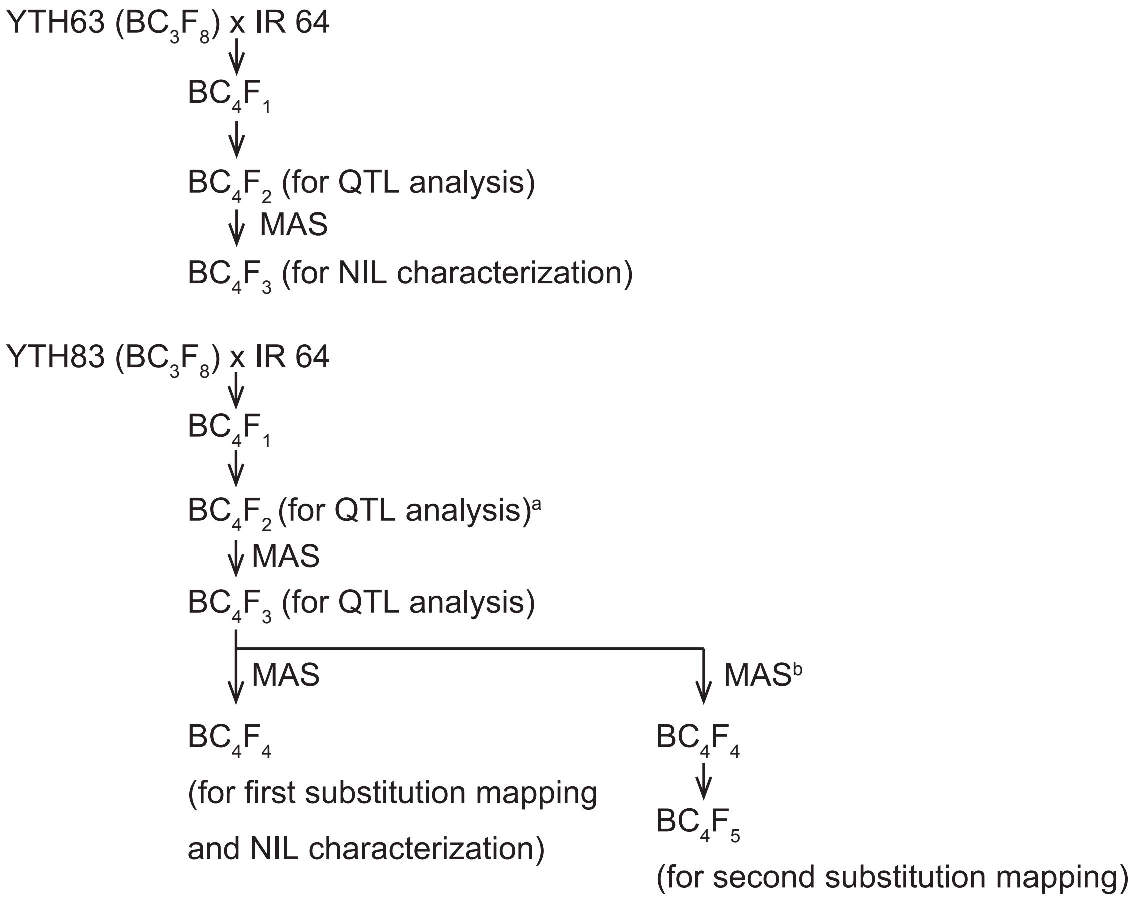

Supplementary Figure S2. Scheme of development for mapping populations and near-isogenic lines.

MAS stands for marker assisted selection.

[a] In this BC_4F_2 population of YTH83 and IR 64, a QTL peak was also found on chromosome 4. We selected a BC_4F_2 plant that was heterozygous on chromosome 12 and IR 64-fixed homozygous on chromosome 4, and BC_4F_3 plants were generated by selfing selected the BC_4F_2 plants and used for QTL analysis.

[b] Different individual plants were used.

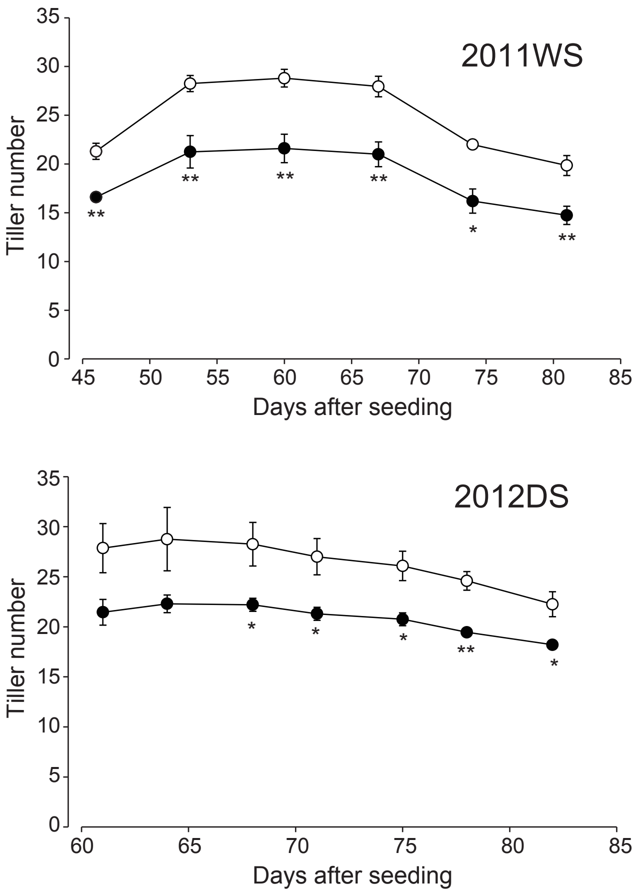

Supplementary Figure S3. Tiller numbers for IR 64 and IR 64-NIL12 grown in a paddy field in the wet season of 2011 and dry season of 2012.
Open circles, IR64; closed circles, IR 64-NIL12. Values from four plots (five hills were measured per plot) were averaged; the average and standard error are shown.
* or ** indicate that IR 64-NIL12 differed significantly from IR 64 at the 5% or 1% level (*t*-test).
