# Supplementary material for: Fine mapping of a quantitative trait locus for spikelet number per panicle in a new plant type rice and evaluation of a near-isogenic line for grain productivity
Source: J Exp Bot. 2017 Jun 3;68(11):2693–702. doi: 10.1093/jxb/erx128 (PMC5853308; doi:10.1093/jxb/erx128)
Supplement: Supplementary_Tables_S1_S3 [file erx128_suppl_supplementary_tables_s1_s3.pdf]

Supplementary TableS1. Molecular markers developed for substitution mapping.

Name	Forward (5´- 3´)	Reverse (5´- 3´)
KM12016	CCCATCGAAGAAAGATGTGG	AGCCTTGCTCAGTGGATCTC
KM12018	CCCATCCGGCCCTAGATTA	TCCGTCCCATAGAAACCAAG
KM12020	ACAGGACCCATCAAGCTCAG	ACAGAGGGAGTGCACGAGAT
KM12021	TTGGTGGCTCGAGATTCTG	TGCACTAGTCACTTTGGCTTCT
KM12022	CCATTCGATTCAATTTGCTG	TGGCCCTACATACCCTCAGT

Supplementary Table S2. Total spikelet number per panicle for IR 64 and IR 64-NIL12 in the experimental field of NARO, Japan, in 2011 and 2012.

Line	2011		2012	
IR 64	124.0 ± 6.5		100.9 ± 3.2	
IR 64-NIL12	149.9 ± 5.4	**	140.1 ± 5.4	***

[a] One panicle at the main stem was sampled per plant. A total of ten panicles in each genotype were investigated; the average and standard error are shown.

[b] All means for IR 64-NIL12 differed significantly from those for IR 64 at 1 % (**) or 0.1 % (***) levels (*t* -test).

Supplementary Table S3. Number of spikelets on primary and secondary branches in IR 64 and near-isogenic lines carrying *qTSN12* in the dry season of 2009.

Line	QTL	Number of spikelet per branch[a]			
		Primary		Secondary	
IR 64	-	5.0 ± 0.1		3.3 ± 0.1	
IR 64-NIL1	*qTSN12.1*	5.0 ± 0.1	ns[b]	3.5 ± 0.0	*
IR 64-NIL12	*qTSN12.2*	5.0 ± 0.1	ns	3.7 ± 0.1	***

[a] At least nine plants in each line were investigated; the average and standard error are shown.

[b] * or *** indicate the NIL differed significantly from IR 64 at the 5 or 0.1 % level (Dunnett's test). ns indicates that the differences were not significant.
